# Quantitative susceptibility mapping (QSM) evaluation of infantile neuroaxonal dystrophy

**DOI:** 10.1259/bjrcr.20180078

**Published:** 2019-02-21

**Authors:** Takuya Fujiwara, Yoshiyuki Watanabe, Hisashi Tanaka, Hiroto Takahashi, Shin Nabatame, Wang Yi, Noriyuki Tomiyama

**Affiliations:** 1 Diagnostic and Interventional Radiology, Osaka University Graduate School of Medicine, Suita, Japan; 2 Department of Pediatrics, Osaka University Graduate School of Medicine, Suita, Japan; 3 Biomedical Engineering, Cornell University, Ithaca, NY, USA

## Abstract

We present the first case of twins with infantile neuroaxonal dystrophy evaluating brain iron deposition using quantitative susceptibility mapping (QSM). A 6-year-old boy who was normal at birth had psychomotor regression and hypotonia from 2-years-old. Brain MRI showed low intensity areas in globus pallidus (GP) and substantia nigra (SN) on *T*_2_* weighted imaging. QSM values of GP and SN were 0.19 and 0.29 ppm, respectively. His twin brother showed almost the same imaging findings. Follow-up MRI revealed increase of QSM value in GP and SN.

## Introduction

Infantile neuroaxonal dystrophy (INAD) is a neurodegenerative condition with brain iron accumulation (NBIA). Patients with INAD typically exhibit psychomotor regression and hypotonia before the age of 2 years and develop pyramidal and extrapyramidal features at a later stage; the neurological prognosis of these patients is poor. Genetically, 80% of children with INAD have mutations in PLA2G6.^1^

Radiologically, the globus pallidus (GP) and substantia nigra (SN) of INAD patients appear hypointense on *T*_2_ and *T*_2_* weighted MRI, which indicates abnormal iron deposition. Cerebellar atrophy can also be observed and it typically precedes iron deposition. Notably, MRI may be normal at the early stage, and radiological findings and symptoms do not always correlate on conventional MRI.^2^

Quantitative susceptibility mapping (QSM) can be used to evaluate the behavior of biomarkers, including iron, calcium, and myelin. To the best of our knowledge, the assessment of INAD using QSM has never been reported; therefore, we believe it is valuable to present the results of our evaluation of QSM in a pair of twins with INAD.

## Clinical presentation

6-year-old monozygotic twin boys were born at term with no complications. They presented with psychomotor regression and hypotonia at 2 years old, spasticity at 4 years old, and ataxia at 5 years old. Both patients had almost the same clinical course. Laboratory results were as follows: blood levels of pyruvic acid and lactate were normal, cerebrospinal fluid analysis was negative for Human T-lymphotropic virus one antibody, and direct sequencing of the PLA2G6 gene revealed c.517*C*>T mutation in p.Q173X transition and c.1634*A*>G mutation in p.K545R transition. They were diagnosed with infantile-onset PLA2G6-associated neurodegeneration, also known as INAD.

## Imaging findings

Brain imaging was performed using a 3 T MRI scanner (Discovery MR750W, GE Healthcare, Milwaukee, WI). Patients underwent *T*_1_-, *T*_2_-, and *T*_2_* weighted imaging. Three-dimensional (3D) gradient multiecho was performed for QSM reconstruction with *T*_1_, *T*_2_, and *T*_2_* weighted images. 3D gradient multiecho was performed for QSM reconstruction with repetition time (TR) = 53.4 ms, echo time (TE) = 13 ms, 17.9 ms, 22.9 ms, 27.8 ms, 32.8 ms, flip angle = 10°, slice thickness = 1.2 mm, 136 slices, band width = 244 Hz/pixel, field of view (FOV) = 24 × 24 cm, matrix size = 384 × 256. QSM images were reconstructed using the morphology enabled dipole inversion method.^3,4^

**Figure 1.**
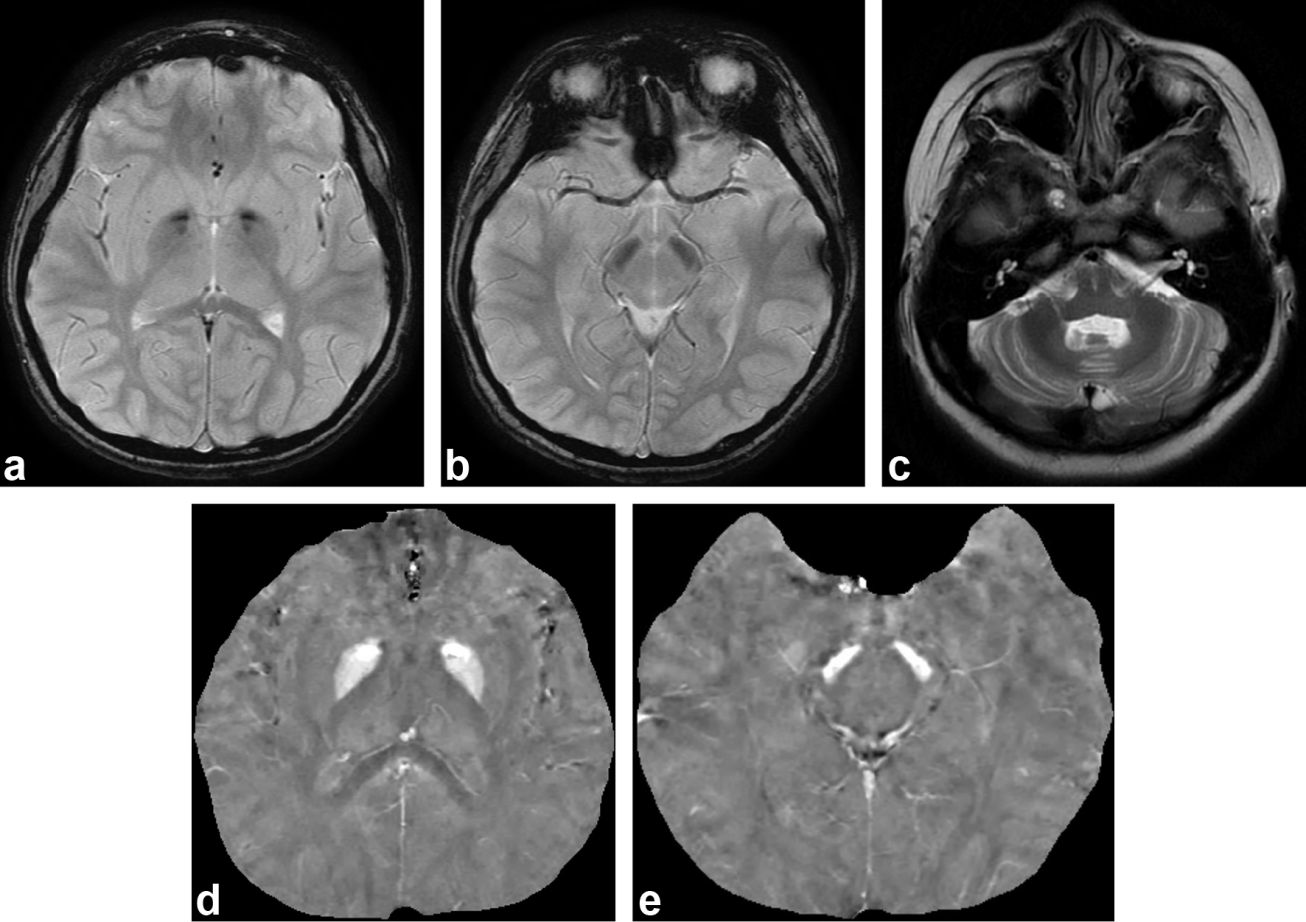
(a, b) *T*_2_* weighted images show hypointensity in GP and SN. (c) Mild cerebellar atrophy was detected on *T*_2_ weighted image. (d, e) GP and SN were hyperintense on quantitative susceptibility mapping. GP, globus pallidus; SN, substantia nigra.

To obtain QSM values in the GP and SN, multiple slices were manually outlined to cover each structure entirely. We also placed regions of interest in the white matter of the occipital lobe and pons that appeared normal on *T*_2_ weighted images as a reference. *T*_2_ weighted (Figure 1a,b) images showed diffuse mild hypointensity in the GP and SN. The most hypointense areas were located in the ventromedial aspect of the GP bilaterally. Mild cerebellar atrophy was also detected; however, the cerebellum showed normal signal intensity (Figure 1c). Cerebral white matter abnormalities and hypertrophy of the clava were not present in these subjects.

Both twins had almost the same findings on MRI, including identical QSM values. Those values were 0.19 ppm in the GP and 0.29 ppm in the SN (Figure 1d,e). We compared the QSM values in the GP and SN from the twins with values from three normal subjects (8–12 years old). The control subjects showed lower QSM values than the twins. We re-evaluated our subjects after 27 months, and the GP and SN were even more hypointense on *T*_2_* weighted images. During that period, the QSM values increased to 0.25 ppm in the GP and 0.33 ppm in the SN (Table 1).

**Table 1.** QSM values (ppm) in the patients with INAD and normal children

	Globus pallidus	Substantia nigra	White matter (occipital lobe)	Pons
Patient 1 (6 y.o.)	0.19	0.29	−0.013	−0.033
Patient 1 (8 y.o.)	0.25	0.34	−0.013	−0.033
Patient 2 (6 y.o.)	0.19	0.29	−0.013	−0.031
Patient 2 (8 y.o.)	0.25	0.31	−0.013	−0.031
Normal 1 (8 y.o.)	0.10	0.10	−0.016	−0.032
Normal 2 (8 y.o.)	0.10	0.08	−0.020	−0.031
Normal 3 (12 y.o.)	0.09	0.11	−0.010	−0.036

INAD, infantile neuroaxonal dystrophy; QSM, quantitative susceptibility mapping.

## Discussion

Our cases showed higher QSM values in the SN and GP than do normal children. In normal subjects, cerebral iron deposition increases with age, and some reports have shown correlations between age and susceptibility measured by QSM.^5–7^ Darki evaluated 21 normal children (age: 6.73 ± 0.27, male/female: 12/8) using QSM. In that study, the average QSM values were 0.05 ppm in the GP and 0.04 ppm in the SN.^8^

Quantitative imaging is an important tool that can be used as a biomarker for early diagnosis, prognostic prediction, and therapy evaluation.^9^ Brain iron deposition has been quantified using QSM in patients with neurodegenerative diseases, such as Parkinson’s disease and multiple sclerosis, as well as other less common conditions.^10,11^ For example, Ishiyama et al measured QSM values in two patients with beta-propeller protein-associated neurodegeneration.^12^ QSM values in the GP and SN were 0.430 and 0.540 ppm in Patient 1 and 0.381 ppm and 0.425 ppm in Patient 2, respectively, and all values were higher than those for controls. It has also been reported that two patients with pantothenate kinase-associated neuropathy and PANK2 mutations have high QSM values in the GP and SN.^13^

Our results reveal that QSM can be used to evaluate iron deposition in patients with INAD. More detailed studies are required to evaluate the correlation between the QSM value and symptoms of efficacy of therapy.

## Learning points

1. INAD is a rare disease that causes iron deposition in the brain. On MRI, *T*_2_ or *T*_2_* hypointensity of the GP and SN are the most common characteristics, while cerebellar atrophy, cerebral and cerebellar white matter abnormalities, and hypertrophy of the clava may be observed.
2. QSM showed higher iron content in the GP and SN in patients with INAD than in normal subjects, and follow-up MRI showed an increase in QSM values due to iron deposition. Therefore, QSM could be used for the quantitative assessment of disease progression and therapy response in the future.
